# Service Profile of Orthognathic Surgery of a Medical School

**DOI:** 10.1590/S1808-86942010000500011

**Published:** 2015-10-22

**Authors:** Caroline Gabriele Marques, José Victor Maniglia, Fernando Drimel Molina

**Affiliations:** 1 Doctoral degree, orthodontist, ENT Department, FAMERP; 2 Associate professor, secretary of health, São José do Rio Preto; 3 Doctoral degree in health sciences, head of the ENT Department at the São José do Rio Preto medical school. São José do Rio Preto Medical School - FAMERP

**Keywords:** sleep apnea, surgery, obstructive.

## Abstract

**Abstract:**

Orthognathic surgeries are very important for both the correction of dentofacial deformities as well as for the treatment of obstructive sleep apnea/hypopnea syndrome. Nowadays, most of the population presents some type of morphological and/or functional disorder of the stomatognathic system.

**Aim:**

The present study aims at assessing the information from the individuals treated in the Orthognathic Surgical Service of a Medical School.

**Methods:**

Search in medical records - 2004-2008, identification procedures, individual characteristics, malocclusion and surgery.

**Results:**

The number of surgical treatments due to dentofacial deformities has been increasing. Maxillary advancement surgery alone comprised the highest number in the sample.

**Conclusion:**

Orthognathic surgery cases have been increasing in the last years and maxillary advancement alone comprised the highest number of surgical treatments.

## INTRODUCTION

Orthognathic surgery is important for correcting dental and facial anomalies and for treating the obstructive sleep apnea and hypopnea syndrome (OSAHS).^1^

These procedures are among the modality of maxillofacial surgery that have developed most in recent years because of faster techniques that decrease morbidity.^2^

Using this type of surgery for correcting malocclusion is not new. The first known osteotomy for this purpose was done by Simon H. Hullihen in 1849, in Wheeling,

West Virginia.^3^

Angle^4^ stated that the only possibility of treating true dentofacial deformities was to combine orthodontics with surgery; he recognized the importance of combining efforts in these two approaches of dentofacial disharmony in patients with skeletal conditions.

Planning should be such that the first step is an analysis of the patient's face aiming at full correction: cosmetic defects and occlusion keys.^5^

Currently we find that most of the population has some type of morphological and/or functional change in the stomatognathic system.^6^ A growing number of dentofacial deformity correction centers in our country and a significantly higher demand for such services has raised the need for studying the prevalence of these deformities to plan and implement adequate treatment.^7^

An analysis by Silva Filho^8^ showed that 88.5% of the population has some degree of occlusal disharmony. Among malocclusions, the most prevalent was cl I (55%), followed by cl II (42%) and cl III (3%).

Obstructive sleep apnea occurs when the passage of air through upper airways is hindered and breathing effort occurs during more than 10 seconds. Hypopnea refers to a decreased air flow in upper airways for at least 10 seconds. These events occur repeatedly and only during sleep, resulting in the typical signs and symptoms of OSAHS.^9^

The OSAHS is a fairly frequent complex and multifactor condition that has been the focus of several specialties. It originates with recurring upper airway obstruction during sleep. Interrupted air flow during more than 10 seconds may result in several symptoms, such as daytime drowsiness, systemic and pulmonary arterial hypertension, arrhythmias, fragmented sleep, and sudden death. This condition is a serious public health issue, which justifies the need for an early diagnosis and prompt therapy.^10^

Facilitating conditions for OSAHS are loss of palatal, tongue and pharyngeal muscle tone, soft tissue collapse over airways secondary to macroglossia, retrognathia and micrognathia, excessive musical folds, excessive submucosal fat, and obstructed nasal pathways.^11^

Lowe et al.^12^ has stated that OSAHS patients present several atypical craniofacial features, such as retropositioned jaws, disorganized occlusal planes, protrusion of incisive teeth, an obtuse gonial angle, and an increased lower facial third height with a tendency for an anterior open bite; this in turn may be associated with a larger tongue with an altered tone, which may also be retropositioned next to the posterior pharyngeal wall.^13^

**Figure 1:**
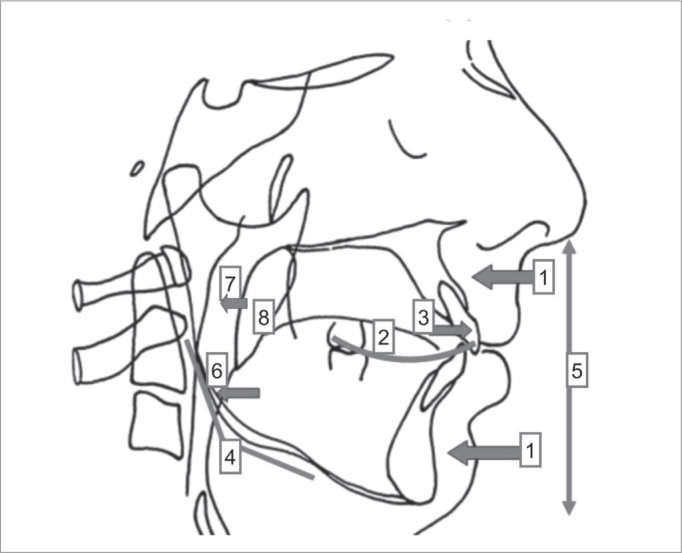
Cephalogram showing the typical facial features of OSAHS subjects.

Dentoskeletal and soft tissue alterations that commonly affect OSAHS patients are (Figure 1):

1 retropositioned jaws;
2 altered Spee curve and disorganized occlusal plane;
3 protrusion of incisives;
4 tendency for vertical facial growth - obtuse gonial angle;
5 increased anterior/inferior facial height;
6 retrolingual collapse;
7 retropalatal collapse;
8 increased soft palate length.

Cephalometric radiography aims to reproduce facial proportions in a standardized manner, expressed in linear and angular measures. Its use in assessing facial morphology has been amply studied (lateral, frontal and basal incidences). Available computerized cephalometric tracings are a valuable aid for standardizing and storing data, and for comparing results with normal reference values.^14^

Neau et al.^15^ have shown that the OSAHS is an independent risk factor for strokes, and should be treated in all stroke patients. These authors have also shown that subjects with a respiratory disorder index over 20 have a higher mortality rate because of cerebrovascular diseases compared to subjects with a score below 20, which becomes even more evident over the age 50 years.^9^

A complete nocturnal polysomnography is the standard test for evaluating sleep-related respiratory disorders. It is mandatory for confirming the presence and severity of these disorders. The most important variables indicating the severity of obstruction include the apnea/hypopnea rate, the respiratory disorder rate, and the saturation of oxyhemoglobin.^16^,^17^

The most common conservative treatments recognized as effective are: nasal continuous positive air pressure (nasal CPAP), oral appliances, and weight loss. Nasal CPAP does not treat the underlying cause, and is indicated for lifelong use.^16^

Riley^18^ (1989) created a surgical protocol for the treatment of OSAHS, which comprises two phases.

Phase I focuses on specific obstructions. Patients with a soft palate obstruction only undergo procedures at this level (uvulopalatopharyngoplasty), and patients with base of tongue obstructions undergo surgery at this anatomical site (mandible osteotomy and genioglossal advancement and/or hyoid bone suspension myotomy. If nasal block is present, it is corrected in phase I.

Phase II includes osteotomy and maxillomandibular advancement surgery, base of tongue surgery, and lingual tonsillectomy.^18^

The purpose of this study was to characterize subjects treated at the Orthognathic Surgery Unit of the Otorhinolaryngology and Head & Neck Surgery Unit of a medical school.

## MATERIAL AND METHODS

The method consisted of an inductive approach with statistical and descriptive procedures. An indirect document review of registries was made for patients seen from 2004 to 2008 at the outpatient clinic of the Orthognathic Surgery Unit of the Otorhinolaryngology and Head & Neck Surgery Unit of a medical school. The survey listed the number of procedures, individual features, the type of malocclusion, and the type of surgery for correcting dentofacial deformities and/or OSAHS.

## RESULTS

Table 1, Table 2, Table 3, Table 4, Table 5

**Table 1:** Distribution by percentages of deformities observed in the sample compared to those observed in the population.

Deformity	No. Sample	% Observed in the Sample	Prevalence in the Population (%)
Cl I	5	7	55
Cl II	24	35	42
Cl III	39	58	3

**Table 2:** Bone substrate and number of treatments of the bone substrates.

Treated Bone Substrate	Number of Treatments
Mandible only	10
Maxilla and mandible	14
Mandible and mentum	1
Mandible, maxilla and mentum	2
Maxilla only	38
Mentum only	3
To t a l	68

**Table 3:** Gender distribution of dentofacial deformities.

Gender	Total
Male	33
Female	35

**Table 4:** Gender distribution of treated dentofacial deformities correlated with the bone substrate involved, the number and the percentage.

Base Óssea					
	Masculino		Feminino		Total
	N	%	N	%	%
Mandíbula exclusivamente	3	9	7	20	15
Maxila/ Mandíbula	13	39,5	1	3	20,5
Mandíbula/ Mento	0	0	1	3	1,5
Mandíbula/ maxila/ Mento	0	0	2	6	3
Maxila exclusivamente	16	48,5	22	62	56
Mento exclusivamente	1	3	2	6	4
Total	33	100	35	100	100

**Table 5:** Distribution of dentofacial deformities treated from 2004 to 2008.

Year	Total
2003	3
2004	6
2005	8
2006	17
2007	13
2008	21

## DISCUSSION

The treatment of dentofacial deformities is currently one of the most debated topics in buccomaxillofacial and craniomaxillofacial surgery. It involves issues in biology, pathophysiology, surgical techniques, anesthesia, pre- and postoperative management, craniofacial growth and development, and facial cosmetics and harmony.

The need to develop dentofacial deformity correction centers in Brazil is significant, given the increased demand for these services, as evidenced in this study during the study period. These data underline the need for constant scientific and technical updates and an understanding of the patients for more an objective approach to dealing with these deformities.

The sample selection in this study was based on an analysis of the registries of treated patients with the aim of assessing the profile of the unit.

The sample comprised 68 Brazilian white adult subjects of both sexes; the mean age was 28.7 years (14-49 years). The underlying feature of these subjects was to present a dentoskeletal deformity. The main complaints were: cosmetic, functional (chewing) and/or OSAHS demonstrated by polysomnography.

Obstructive sleep apnea occurs when the passage of air through upper airways is hindered and breathing effort occurs during more than 10 seconds. Hypopnea refers to a decreased air flow in upper airways for at least 10 seconds. These events occur repeatedly and only during sleep, resulting in the typical signs and symptoms of OSAHS.^9^

The male-female distribution in the sample was fairly balanced; this finding differs from most of the reviewed published studies on patients referred for dental-surgical therapy^19^,^20^,^21^ in that generally there are more females than males, because of a more refined perception of the self-image and greater compliance with this type of therapy in women.

Orthognathic surgery aims to treat dentofacial deformities; it is important not only for correcting occlusion but also for facial cosmetics. Thus, psychosocial aspects relate directly with this form of therapy, since the appearance of the face affects body image formation, identity and self-esteem.^22^ This may explain why there are more female patients in orthognathic surgery units; differing from our findings in that the number of males and females is fairly similar. An explanation is that orthognathic surgery patients seek not only cosmetic-functional treatment but also therapy for the OSAHS, which is more frequent in males.

There were more Angle class III patients in our sample, which differs from the study of Silva Filho, in which the cl I malocclusion was more prevalent.

Our Unit treats dentofacial and skeletal deformities. It is possible that Angle cl III patients had significant cosmetic and functional complaints difficult to treat orthodontically only. Thus, such cases are more frequent at our Unit, differing from prevalence studies in the general population, in which there are more Angle cl I individuals.

The number of subjects that presented at our Unit for the treatment of the Angle cl II condition is similar to that in other population studies.

An important finding in our study was that the maxilla was operated alone in 56% of patients, and the maxilla and the mandible were operated in 20.5% of patients, comprising 76.5% of the study sample. We found that many malocclusion patients had maxillary and mandibulary conditions, which require an accurate and detailed diagnosis of malocclusion and the face. All affected anatomical structures should be taken into account separately and jointly for successful treatment involving the occlusal and cosmetic and functional aspects to provide skeletal and dental stability, and muscle and respiratory function, so that retropalatal and retrolingual collapse be treated in OSAHS patients.

## CONCLUSION

The number of surgical procedures for correcting deformities has increased. Although the cl II deformity is the most prevalent in the population, these were not the most frequently treated deformity by surgery. Most cases were maxillary only advancement surgery.
